# Multiomic analysis of the synthetic pathways of secondary metabolites in tobacco leaves at different developmental stages

**DOI:** 10.3389/fpls.2025.1615756

**Published:** 2025-06-24

**Authors:** Zhijun Tong, Kun Yang, Xuejun Chen, Fei Xu, Xueyi Sui, Yujie Huang, Shenyun Zhu, Enhui Shen, Sanling Wu, Longjiang Fan, Bingguang Xiao

**Affiliations:** ^1^ Key Laboratory of Tobacco Biotechnological Breeding, Yunnan Academy of Tobacco Agricultural Sciences, Kunming, China; ^2^ Institute of Crop Sciences & Institute of Bioinformatics, College of Agriculture and Biotechnology, Zhejiang University, Hangzhou, China; ^3^ Analysis Center of Agrobiology and Environmental Sciences, Faculty of Agriculture, Life and Environment Sciences, Zhejiang University, Hangzhou, China

**Keywords:** tobacco, multiomic analysis, secondary metabolites, developmental stages, transcriptomic

## Abstract

**Introduction:**

*Nicotiana tabacum*, widely cultivated for its economic and scientific value, produces a broad range of secondary metabolites that play critical roles in determining leaf quality and flavor. Despite substantial progress, the comprehensive regulatory landscape governing secondary metabolite biosynthesis during *N. tabacum* leaf development remains largely unclear.

**Methods:**

To better understand the molecular regulatory mechanisms underlying the biosynthesis of secondary metabolites, particularly flavonoids, during *N. tabacum* leaf development, we conducted a transcriptomic and non-targeted metabolomic sequencing and analysis at three critical developmental stages: vigorous growth stage (T1), topping stage (T2), and harvest stage (T3).

**Results:**

Based on our transcriptomic and metabolomic data, 25 unigenes exhibiting stage-specific expression patterns that were strongly associated with flavonoid accumulation were identified. We found that during early developmental stages (T1-T2), upregulated expression of chalcone synthase (CHS) and chalcone isomerase (CHI) correlated with enhanced flavonoid backbone biosynthesis. In contrast, during the later stage (T3), increased expression of dihydroflavonol 4-reductase (DFR) and anthocyanidin synthase (ANS) was consistent with elevated anthocyanin accumulation.

**Conclusion:**

This study systematically analyzed the coordinated regulatory network of flavonoid biosynthesis during leaf development in *N. tabacum*, revealing dynamic metabolic shifts across developmental stages. The findings offer novel molecular insights into the mechanisms underlying leaf quality formation and establish a theoretical framework for functional studies of candidate genes, reinforcing the utility of *N. tabacum* as a model species for secondary metabolism research and breeding innovation.

## 1 Introduction

Tobacco (*Nicotiana tabacum* L.), a significant economic crop in the Solanaceae family, originated in the Americas and is now extensively cultivated in Cuba, China, the United States, and other regions. Beyond its commercial value, tobacco serves as an important model organism for studying the regulatory mechanisms of plant secondary metabolism (Jassbi et al., 2017). The plant produces diverse secondary metabolites, including alkaloids, phenolic compounds, and terpenoids, which play pivotal roles in plant defense (Yan et al., 2017; Elser et al., 2023), stress adaptation (Hu et al., 2023; Gu et al., 2024), and environmental interactions (Wu et al., 2025). While nicotine and related alkaloids have been the primary focus of previous research, metabolites such as flavonoids, polyamines, and diterpenes also contribute significantly to the chemical diversity and biological functionality of tobacco leaves (Wu et al., 2023). These compounds not only participate in plant defense and environmental adaptation, but also play key roles in determining tobacco leaf quality and flavor (Wang et al., 2024).

The biosynthesis of secondary metabolites is regulated at multiple levels, exhibiting distinct dynamic patterns during leaf development (Qin et al., 2020; Lv et al., 2024). Using an integrated metabolomics approach, we can characterize the dynamic changes of metabolites and bioactivities during plant leaf development (Bai et al., 2024). The changes in metabolic profiles during leaf growth and development accurately reflect alterations in plant physiological functions, energy metabolism requirements, and environmental adaptation strategies (Zhang et al., 2018). For instance, young leaves are primarily characterized by active cell division and primary metabolic activities, while mature leaves tend to accumulate secondary metabolites associated with tissue differentiation and environmental defense functions (Zhou and Liu, 2022).

The application of multi-omics technologies has become a widely adopted method for exploring intricate biosynthetic mechanisms. Multi-dimensional data analysis enables in-depth exploration of the intricate regulatory networks governing secondary metabolite biosynthesis. Transcriptomics can precisely reflect gene expression patterns, comprehensively revealing the dynamics of gene expression in metabolic pathways and providing a crucial foundation for identifying key structural genes and transcription factors. For example, transcriptomic analysis of cold-stressed tobacco revealed 16,204 differentially expressed genes and highlighted the role of photosynthesis and flavonoid biosynthesis pathways (Song et al., 2024). Metabolomics technologies, particularly untargeted metabolomics, can directly capture end-product information of gene expression, comprehensively detecting metabolite species and providing evidence for further elucidating the mechanisms underlying phenotypic formation (Zhang et al., 2022). By integrating transcriptomics and metabolomics, researchers can systematically investigate the relationship between transcriptional regulatory networks and metabolite biosynthesis during plant development (Xu et al., 2020; Yang et al., 2023a). Through an integrative analysis of transcriptomic and metabolite profiles (Wang et al., 2023), demonstrated that phosphate application enhanced cold tolerance in alfalfa by modulating the biosynthesis of key metabolites. Similarly, Moschen et al. identified transcription factors that were pregulated under drought conditions in sunflower, enhanced understanding of the molecular mechanisms involved in sunflower under drought conditions (Moschen et al., 2017).

In tobacco research, integrated multi-omics analyses have successfully identified stage-specific metabolic pathways and candidate regulatory genes involved in nicotine biosynthesis, flavonoid modification, and terpenoid diversification, which deepens our understanding of the complex regulatory networks underlying tobacco leaf development and secondary metabolism (Jiao et al., 2020; Yang et al., 2023b; Niu et al., 2025). These findings not only reveal the dynamic coordination between metabolic flux and gene expression but also provide potential molecular targets for variety improvement and metabolic engineering (Qi et al., 2021; Pan et al., 2025). Moreover, a deeper understanding of the developmental regulation of secondary metabolism offers valuable guidance for optimizing leaf harvesting timing, curing methods, and processing techniques. As tobacco continues to serve as a model for studying specialized metabolism, such omics-driven insights will also benefit the broader plant science community by informing research on stress adaptation, metabolite regulation, and biomass quality improvement.

Despite significant progress, the complete regulatory landscape of secondary metabolite biosynthesis during tobacco leaf development remains to be fully elucidated (Chang et al., 2020). The complexity arises from the diversity of metabolites, spatiotemporal specificity of gene expression, and the confounding effects of environmental factors (Hu et al., 2024). Therefore, systematically integrating transcriptomic and metabolomic data across critical developmental stages offers valuable insights into the regulation of secondary metabolism in model plant systems, and provides a solid foundation for reconstructing comprehensive metabolic networks.

## 2 Materials and methods

### 2.1 Plants and sample preparation

Samples of a flue-cured tobacco cultivar LY09A were collected from the experimental field of the Yunnan Academy of Tobacco Agricultural Sciences, located in Kunming City, Yunnan Province (25°03′N, 102°39′E). Leaf samples were harvested at three key developmental stages: the vigorous growth stage (T1, 60 days after transplanting), the topping stage (T2, 90 days after transplanting), and the harvest stage (T3, 150 days after transplanting).

### 2.2 Metabolite extraction and profiling

Metabolite extraction and profiling were performed by Wuhan Metware Biotechnology Co., Ltd. using non-targeted metabolomics approaches. Briefly, 20 mg of powdered tobacco leaf samples were homogenized with 400 μL of 70% methanol containing internal standards (2-chlorophenylalanine) and vortexed for 15 min. After adding 200 μL petroleum ether, the mixture was vortexed for 5 min and centrifuged at 12,000 ×g for 10 min at 4°C. The lower aqueous phase was filtered through a 0.22-μm PTFE membrane and stored at −20°C prior to LC-MS/MS analysis. Chromatographic separation was achieved on a Waters ACQUITY UPLC HSS T3 C18 column (1.8 μm, 2.1 × 100 mm) maintained at 40°C. The mobile phases consisted of (A) ultrapure water with 0.1% formic acid and (B) acetonitrile with 0.1% formic acid, delivered at 0.4 mL/min with the following gradient: 5% B (0 min), linear increase to 90% B (11 min), held for 1 min, then returned to 5% B (12.1 min) and re-equilibrated for 1.9 min. The injection volume was 2 μL.Mass spectrometry was performed on an Agilent 6545 QTOF/MS system equipped with an ESI source operating in both positive (ESI+) and negative (ESI−) ionization modes. Key parameters included: ion source voltage (± 2,500 V for ESI+, ± 1,500 V for ESI−), nebulizer gas (40 psi), drying gas flow (8 L/min at 325°C), and sheath gas flow (11 L/min at 325°C). Data acquisition covered m/z 50–1,000 with MS/MS fragmentation at 135 V.

Raw data were converted to mzXML format using ProteoWizard (Holman et al., 2014), processed via XCMS for peak alignment, retention time correction, and SVR-based normalization. Metabolites were annotated by matching against in-house and public databases (KEGG), with stringent QC criteria (CV < 30% in QC samples). A total of 8,651 metabolites were detected, including 3,063 with MS/MS verification (2,525 in ESI+, 538 in ESI−).

### 2.3 Whole-transcriptome sequencing and transcriptomic analysis

Samples for RNA sequencing were collected in 2022. Three replicates of each plant were sent to BGI for RNA-seq on the BGISEQ-500 platform and bisulfate-seq. mRNA was purified using poly-T oligo-attached magnetic beads, fragmented at high temperature with divalent cations, and converted to first-strand cDNA with reverse transcriptase and random primers. Second-strand cDNA was synthesized using DNA Polymerase I and RNase H, followed by adapter ligation after adding an ‘A’ base. Products were purified and enriched via PCR amplification. PCR yield was quantified by Qubit, and samples were pooled to form single-stranded DNA circles (ssDNA circles) for the final library. ssDNA circles underwent rolling circle replication (RCR) to generate DNA nanoballs (DNBs), which enhanced fluorescent signals during sequencing (Zhai et al., 2020). DNBs were loaded onto patterned nanoarrays, and 100 bp (or 150 bp) paired-end reads were performed on the BGISEQ-500 platform (Fehlmann et al., 2016) using the Combinational Probe-Anchor Synthesis Sequencing Method for subsequent data analysis.

### 2.4 Statistical analysis

Raw RNA-seq reads were initially quality-filtered and trimmed using fastp (version 0.21.0) (Chen et al., 2018) with default parameters. Clean reads longer than 60 bp and without ambiguous bases (N) were retained for downstream analysis. The filtered reads were then aligned to the Nicotiana tabacum version ZY300 reference genome (https://www.ebi.ac.uk/ena/browser/view/PRJEB85578) using HISAT2 (Kim et al., 2015). Gene expression levels were quantified using StringTie (version 2.1.4) (Pertea et al., 2015) and normalized using two metrics: transcripts per million (TPM) and fragments per kilobase of exon per million mapped fragments (FPKM). Differential expression analysis between different developmental stages was conducted using the DESeq2 (Love et al., 2014), and genes with |log_2_(fold change)| ≥ 1 and adjusted p-value < 0.05 were considered significantly differentially expressed. Functional annotation of differentially expressed and novel genes was performed using BLAST searches against public databases, including Gene Ontology (GO) (The Gene Ontology Consortium, 2021) and the Kyoto Encyclopedia of Genes and Genomes (KEGG), and enrichment analyses were used to identify significantly associated biological processes and metabolic pathways.

## 3 Results

### 3.1 Transcriptomic and metabolomic profiling in tobacco leaves

**Figure 1:**
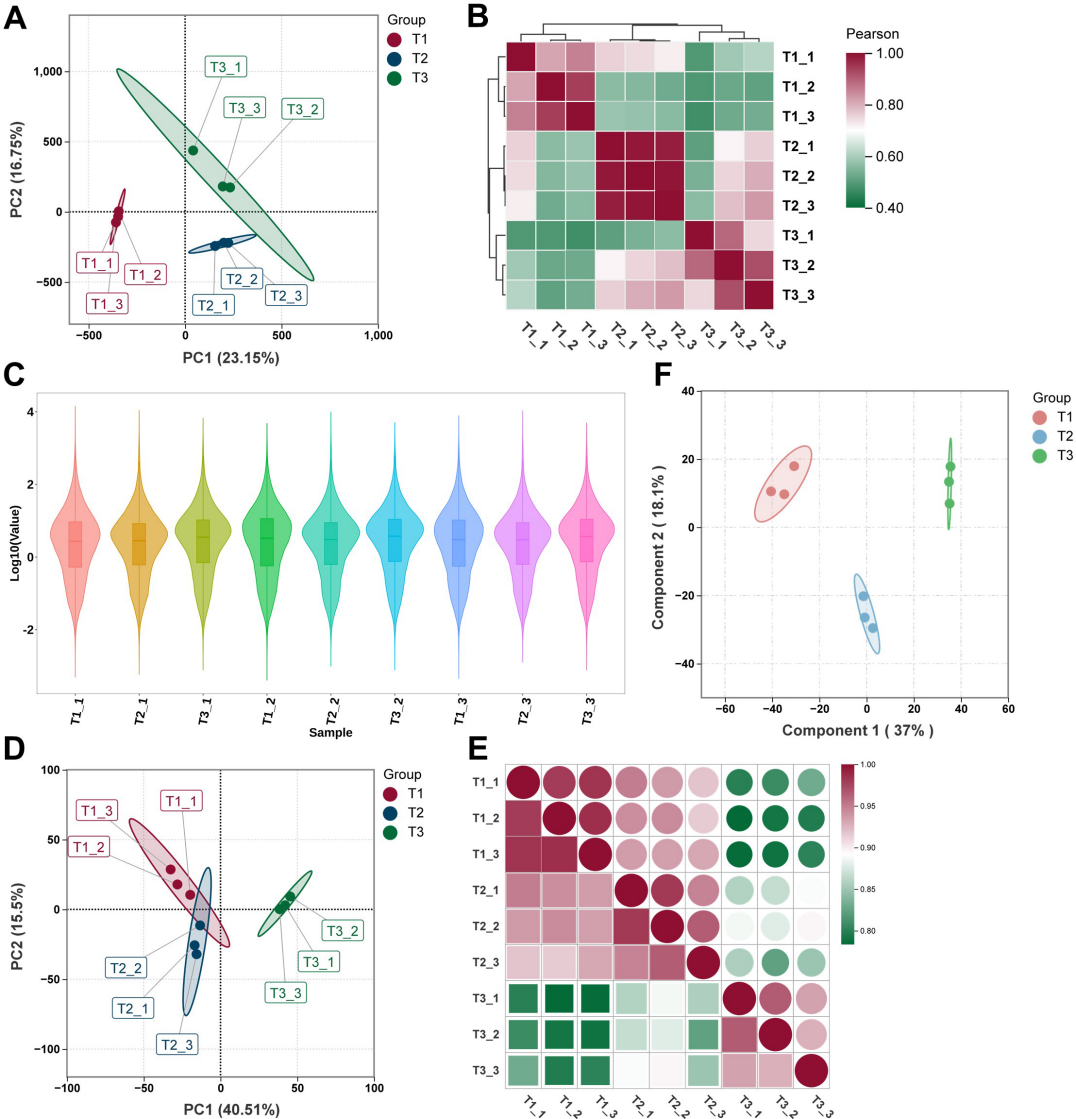
Overview of transcriptomic and metabolomic data of tobacco leaves at different developmental stages. **(A)** Principal component analysis (PCA) of transcriptomic data from T1, T2, and T3 stages. **(B)** Pearson correlation heatmap of transcriptomic data across all biological replicates. **(C)** Violin plots showing the distribution of gene expression values across all transcriptomic samples. **(D)** PCA of untargeted metabolomic data from T1, T2, and T3 stages. **(E)** Pearson correlation heatmap of metabolomic data across all biological replicates. **(F)** Orthogonal partial least squares discriminant analysis (OPLS-DA) score plot showing clear separation of metabolic profiles among the three developmental stages.

After rigorous data processing and quality control, we obtained high-quality transcriptomic datasets (**Supplementary Table S1**). RNA sequencing (RNA-seq) and untargeted metabolomic profiling were employed to systematically investigate transcriptomic and metabolic changes in tobacco leaves across three developmental stages: T1, T2, and T3. Principal component analysis (PCA) revealed distinct stage-specific clustering at both the transcriptomic and metabolomic levels. For the RNA-seq data, principal components 1 and 2 (PC1 and PC2) accounted for 23.15% and 16.75% of the total variance, respectively (**Figure 1A**). In the metabolomic data, PC1 and PC2 explained 40.51% and 16.50% of the variance (**Figure 1D**), highlighting substantial transcriptional and metabolic reprogramming during leaf development. Pearson correlation analysis further confirmed the reliability and consistency of both datasets. Biological replicates from the same developmental stage exhibited strong positive correlations (*r* > 0.9), while correlations between different stages—particularly between T1 and T3—were significantly lower (**Figures 1B, E**). Violin plots of log-transformed gene expression values showed consistent distributions across all RNA-seq samples (**Figure 1C**), indicating uniform sequencing depth and high data quality. To enhance the resolution of metabolomic differences, orthogonal partial least squares discriminant analysis (OPLS-DA) was conducted. This analysis clearly separated the metabolic profiles of the T1, T2, and T3 stages (**Figure 1F**), further supporting distinct metabolic reprogramming during tobacco leaf development.

**Figure 2:**
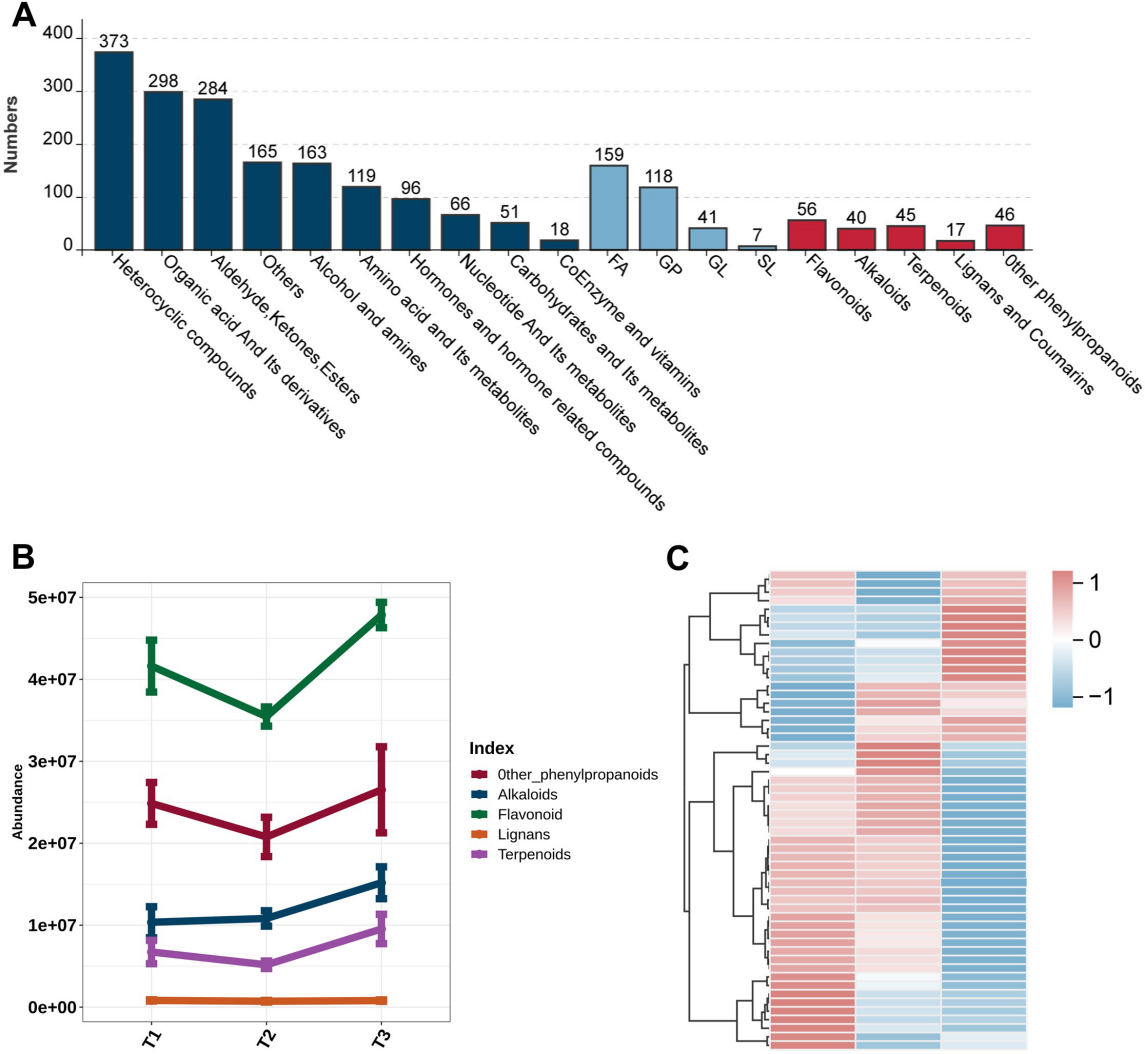
Classification of metabolites identified in tobacco leaves and dynamic changes in secondary metabolite contents. **(A)** Column chart of the classification of metabolites, with the height of different columns representing the number of different compound classes. **(B)** Line chart of the trend of different compound species over time. **(C)** Heatmap of flavonoid metabolite accumulation patterns at different developmental stages.

Meanwhile, we annotated and classified the differentially accumulated metabolites based on their chemical structures and biological functions detected by non-targeted metabolomics technology. In total, over 2,300 high-confidence metabolites were identified, with heterocyclic compounds (373), organic acid derivatives (298), and aldehyde/ketone derivatives (284) representing the three major categories (**Figure 2A**). In addition to primary metabolites, several key secondary metabolites associated with plant growth and defense were also detected, including flavonoids, alkaloids, lignans/coumarins, and terpenoids. Further analysis of their relative abundance revealed distinct accumulation patterns of secondary metabolites across developmental stages: alkaloids increased significantly at the T3 stage, whereas flavonoids and lignans accumulated progressively during leaf maturation (**Figure 2B**). This phenomenon may be attributed to the substantial allocation of nitrogen resources to nicotine synthesis during tobacco leaf maturation, leading to alkaloid accumulation. In contrast, the rise in flavonoid and lignan levels might be linked to enhanced antioxidant capacity and adaptive responses to environmental stress during leaf development (**Supplementary Table S2**) (**Figure 2C**). Collectively, these results demonstrate the rich metabolic diversity of tobacco leaves during development and highlight the dynamic changes in secondary metabolites, particularly flavonoids.

### 3.2 Stage-specific functional pathway shifts during tobacco leaf development

To reveal the stage-specific transcriptomic changes and their causes during tobacco leaf development, differential gene expression (DEG) analysis and KEGG pathway enrichment were performed across three developmental stages (T1, T2, and T3). The results showed a large number of DEGs in each pairwise comparison, with 9,962 genes between T1 and T2, 9,863 genes between T1 and T3, and 4,626 genes between T2 and T3. Notably, more drastic transcriptomic changes were observed between T1 and the later stages (T2 and T3) (**Supplementary Tables S4**-**S6**) (**Supplementary Figures S1A-C**). Further enrichment analysis of the KEGG pathway on DEGs revealed that the biological processes involved in different developmental stages showed significant differences. In the T1 vs. T2 comparison, DEGs were significantly enriched in pathways related to energy metabolism and pigment biosynthesis, such as photosynthesis, carbon fixation, flavonoid biosynthesis, and terpenoid backbone biosynthesis (**Supplementary Figure S1D**). In the T1 vs. T3 comparison, in addition to these pathways, DEGs were also enriched in plant hormone signal transduction, Th17 cell differentiation, and Toll-like receptor signaling, indicating activation of complex hormonal and immune regulatory networks during leaf maturation (**Supplementary Figure S1E**). The T2 vs. T3 comparison revealed enrichment in detoxification and stress response pathways, including cytochrome P450-mediated metabolism, glutathione metabolism, and xenobiotic metabolism. Lipid-related pathways such as linoleic acid metabolism, steroid hormone biosynthesis, and PPAR signaling were also significantly enriched (**Supplementary Figure S1F**). These findings suggest a clear developmental trajectory in tobacco leaves, transitioning from early-stage energy assimilation and pigment production to late-stage stress adaptation and metabolic specialization.

**Figure 3:**
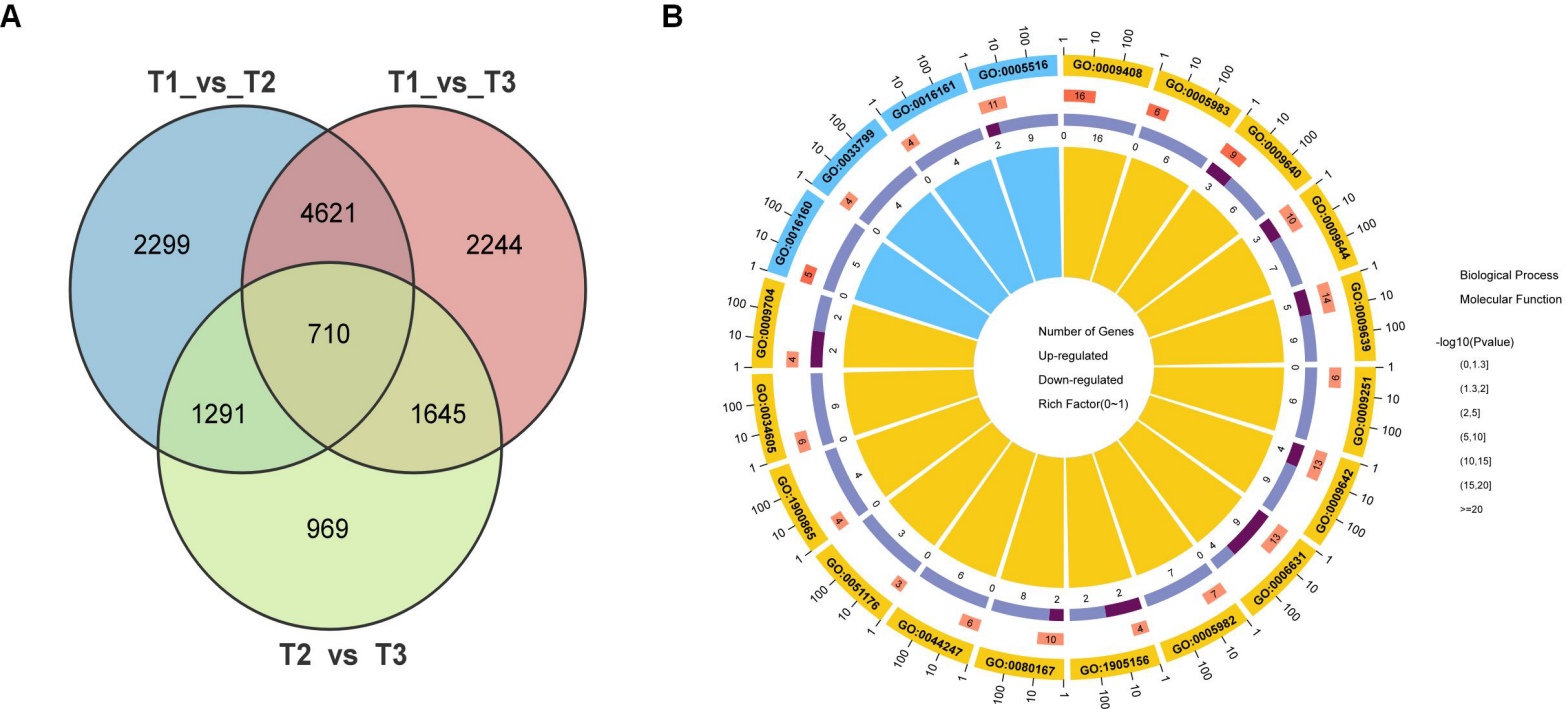
Overlap and functional classification of differentially expressed genes (DEGs) during tobacco leaf development. **(A)** Venn diagram showing the number of DEGs identified in three pairwise comparisons: T1 vs T2, T1 vs T3, and T2 vs T3. A total of 710 DEGs were shared among all three comparisons. **(B)** GO enrichment analysis of the overlapping DEGs.

Venn diagram analysis identified 710 differentially expressed genes (DEGs) that were shared across all three pairwise comparisons (**Figure 3A**), representing a core set of genes dynamically regulated throughout the developmental and maturation stages of tobacco leaves (**Supplementary Table S7**). Gene Ontology (GO) enrichment analysis of these overlapping DEGs revealed significant enrichment in biological processes related to metabolism and response to stimuli, as well as cellular components such as membranes and organelles (**Figure 3B**). These results suggest that common molecular pathways are engaged during various developmental transitions, potentially playing critical roles in determining leaf phenotype and function.

### 3.3 Metabolomic studies revealed differentially accumulated metabolites in tobacco leaves at different developmental stages

**Figure 4:**
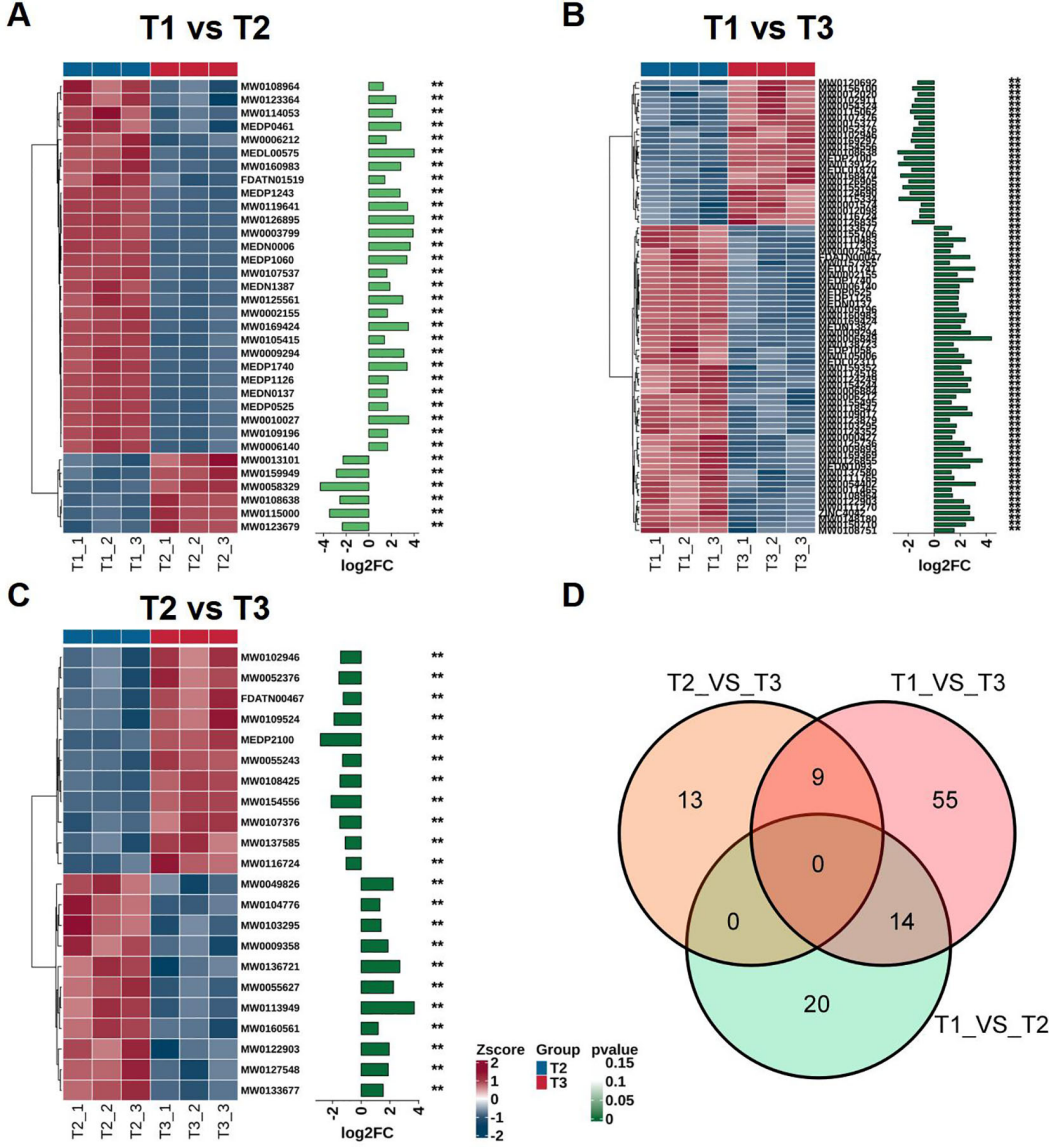
Specific changes at different stages of differentially accumulated metabolites (DAMs) in tobacco leaves. **(A)** T1 and T2 comparison, **(B)** T1 and T3 comparison, **(C)** T2 and T3 comparison, **(D)** Venn diagram illustrating the overlap of DAMs among the three pairwise comparisons. "**" indicates a statistically significant difference at p < 0.01 based on Student's t-test.

In order to study the dynamic changes of metabolites during tobacco leaves development, we compared the metabolomic data of T1, T2, and T3 with each other. A total of 28 differentially accumulated metabolites (DAMs) were identified between T1 and T2, and most showed significant up-accumulation (log2FC > 1, *p* < 0.05) (**Figure 4A**). In the comparison between T1 and T3, more DAMs were detected, indicating that the metabolites changed more significantly with leaves development (**Figure 4B**). It is worth noting that several metabolites showed continuous accumulation from T1 to T3, possibly participating in the metabolic process of leaves maturation. In the comparison of T2 and T3, there were only fewer DAMs, which means that the most significant metabolic changes occurred during the early transition from T1 to T2 (**Figure 4C**). The Venn diagram illustrates the overlaps of DAMs between pairwise comparisons of developmental stages (T1 vs. T2, T1 vs. T3, and T2 vs. T3) (**Figure 4D**). Notably, 14 DAMs were shared between T1 vs. T2 and T1 vs. T3, primarily including amino acids and their derivatives (e.g., O-acetyl-L-serine, L-phenylalanine), benzene derivatives (e.g., oxadixyl, benzaldehyde), nucleotides, and various aldehydes and ketones, indicating their consistent involvement in early to late leaf development transitions. Additionally, 9 DAMs were common between T1 vs. T3 and T2 vs. T3, comprising carbohydrates, organic acids, heterocyclic compounds, steroids, and other metabolites, reflecting metabolic shifts associated with the maturation and harvest stages. The absence of shared DAMs between T1 vs. T2 and T2 vs. T3 comparisons, as well as no metabolites common across all three stages, underscores the stage-specific metabolic remodeling in tobacco leaves, supporting the notion of a tightly regulated biosynthetic network modulating secondary metabolism throughout development. These DAMs are mainly enriched in pathways related to secondary metabolism, including alkaloid biosynthesis, phenylpropane metabolism, and flavonoid biosynthesis, and may play potential roles in regulating tobacco leaves quality and adapting to environmental stresses during development. A detailed list of DAMs and their statistical parameters can be found in **Supplementary Table S8**.

### 3.4 The transcription levels of genes related to the flavonoid biosynthesis pathway at different developmental stages

**Figure 5:**
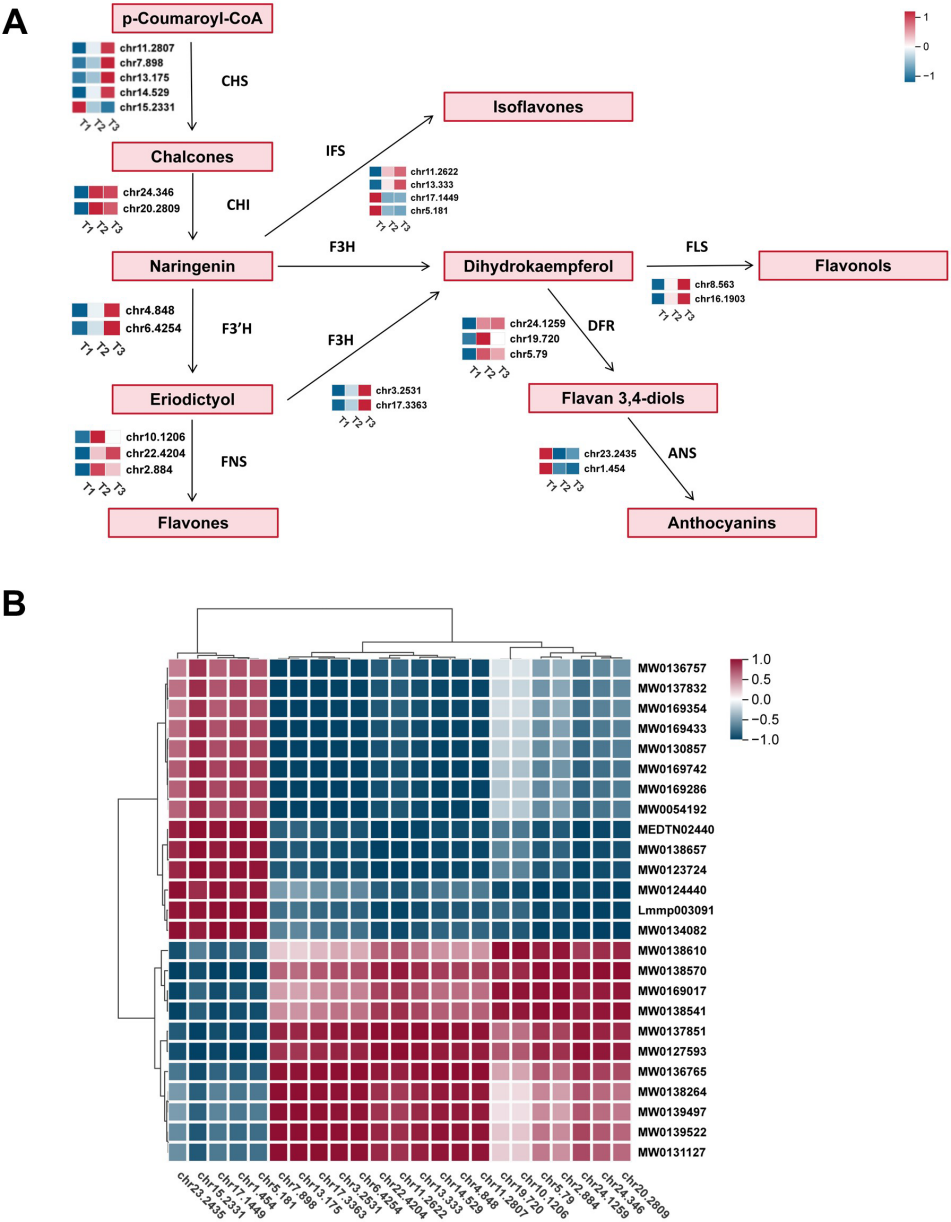
The transcriptional map of flavonoid biosynthesis pathway genes in tobacco across different developmental stages. **(A)** A total of 25 unigenes encoding key enzymes involved in the flavonoid biosynthesis pathway were identified, including CHS, CHI, F3H, F3’H, FNS, IFS, FLS, DFR, and ANS. The heatmaps next to each enzyme represent the relative expression levels of the corresponding unigenes at three developmental stages. **(B)** Hierarchical clustering heatmap of 27 flavonoid biosynthesis-related genes at three developmental stages.

Based on KEGG pathway enrichment and GO functional annotation, a total of 25 unigenes encoding key enzymes involved in the flavonoid biosynthesis pathway were identified in this study. These included five unigenes encoding chalcone synthases (CHS), two for chalcone isomerases (CHI), two for flavanone 3-hydroxylases (F3H), three for dihydroflavonol 4-reductases (DFR), two for flavonol synthases (FLS), and two for anthocyanidin synthases (ANS), among others — all of which play vital roles in catalyzing distinct steps of the flavonoid biosynthetic pathway (**Figure 5A**). Heatmap analysis revealed distinct expression patterns of these genes across the three developmental stages (T1, T2, and T3) (**Figure 5B**). For example, CHS genes (e.g., chr7.3897 and chr7.1255) and CHI genes showed significantly higher expression levels during the early stage (T1), suggesting their critical roles in the initial synthesis of flavonoid backbones. In contrast, downstream genes such as DFR (chr4.7209, chr7.6759) and ANS (chr6.2245, chr7.4145), which are involved in anthocyanin biosynthesis, were markedly upregulated at the late developmental stage (T3), indicating a potential accumulation of anthocyanin compounds during leaf maturation (**Supplementary Table S9**). Overall, these results demonstrate that the transcriptional regulation of flavonoid biosynthesis-related genes is highly stage-specific, with different enzymatic steps activated at specific growth stages to coordinate the metabolic flow of flavonoids. These findings provide important insights into the regulatory mechanisms of flavonoid biosynthesis and the identification of key genes for future research.

## 4 Discussion

In this study, we identified 25 key candidate genes potentially involved in flavonoid metabolism regulation through transcriptomics and non-targeted metabolomics, laying a crucial theoretical foundation for deciphering the flavonoid metabolic network in tobacco leaves. Notably, although this research primarily focused on flavonoids as important secondary metabolites, the significant dynamic changes and biosynthetic mechanisms of other metabolic pathways—such as terpenoids, alkaloids, and phenolic acids—during tobacco growth and development remain worthy of investigation. This is particularly relevant given existing studies demonstrating pronounced changes in alkaloids during tobacco root development. These secondary metabolites may exhibit spatiotemporal coordination with flavonoid biosynthesis, providing valuable insights for further research into their potential roles in regulating tobacco quality, stress responses, and developmental processes. Future studies should expand the analytical scope to elucidate the interactions among different metabolic pathways, thereby achieving a comprehensive, systems-level understanding of metabolic regulation during tobacco development.

Moreover, although the integration of metabolomic and transcriptomic data has enabled preliminary construction of a regulatory network for flavonoid biosynthesis, the universality and robustness of these regulatory mechanisms still require further validation. Transcript-level expression changes alone are insufficient to comprehensively and accurately determine the biological functions of the 25 candidate genes. Therefore, subsequent studies should employ experimental validation approaches, such as gene overexpression and CRISPR/Cas9 genome editing, to elucidate the specific roles of these genes in flavonoid synthesis while further characterizing their core expression patterns.

With the continuous advancement of sequencing technologies, it is imperative to conduct population-scale investigations across diverse tobacco germplasm resources. Strategies such as metabolome-wide association studies (mGWAS), expression quantitative trait loci (eQTL) mapping, and genetic variation analysis can reveal conserved regulatory patterns and core regulatory elements within natural populations. These findings will not only provide theoretical guidance for breeding high-quality tobacco varieties but also yield valuable molecular markers for practical applications.

In summary, this study systematically elucidates the metabolic regulatory mechanisms of flavonoid biosynthesis during tobacco leaf development while highlighting the need for functional validation and population-level studies to fully uncover its biological significance and breeding potential.

In addition to the current findings, there are several valuable directions for future research. For example, the identification of key candidate genes involved in flavonoid biosynthesis provides a valuable starting point for future functional studies and it will be better to perform in-depth functional analyses, including gene editing and overexpression experiments, to uncover the specific regulatory roles of these genes. Furthermore, recognizing the diversity among tobacco varieties—such as flue-cured, cigar, and other types, it is important to explore their metabolomic and transcriptomic differences during similar developmental stages. The integrated their datasets and comparative analyses will help elucidate the molecular basis underlying varietal differences in leaf characteristics and contribute to a broader understanding of metabolic regulation across tobacco types.

## 5 Conclusion

This study employed an integrated transcriptomic and metabolomic approach to investigate the molecular characteristics of flavonoid biosynthesis and associated differential metabolites across different developmental stages of tobacco leaves. A total of 25 unigenes involved in flavonoid biosynthesis were identified, whose expression patterns showed strong correlation with the accumulation profiles of flavonoid metabolites, including flavones, flavonols, and anthocyanins. The early developmental stages (T1-T2) were characterized by upregulated expression of CHS, CHI, and F3H genes, promoting flavonoid skeleton formation. In contrast, the later stage (T3) exhibited a metabolic shift toward anthocyanin biosynthesis, marked by increased expression of DFR and ANS genes. These findings elucidate the coordinated regulatory network between gene expression and metabolite accumulation in secondary metabolism, providing a theoretical foundation for improving tobacco leaf quality through metabolic engineering and precision breeding strategies.

## Data Availability

The transcriptome data presented in this study have been deposited in the National Genomics Data Center (NGDC) under BioProject accession number PRJCA039108, and the raw RNA-seq data are available in the Genome Sequence Archive (GSA) under accession number CRA024963.
