# Supplementary material for: Multiomic analysis of the synthetic pathways of secondary metabolites in tobacco leaves at different developmental stages
Source: Front Plant Sci. 2025 Jun 24;16:1615756. doi: 10.3389/fpls.2025.1615756 (PMC12235415; doi:10.3389/fpls.2025.1615756)
Supplement: Supplementary file 2 [file DataSheet1.pdf]

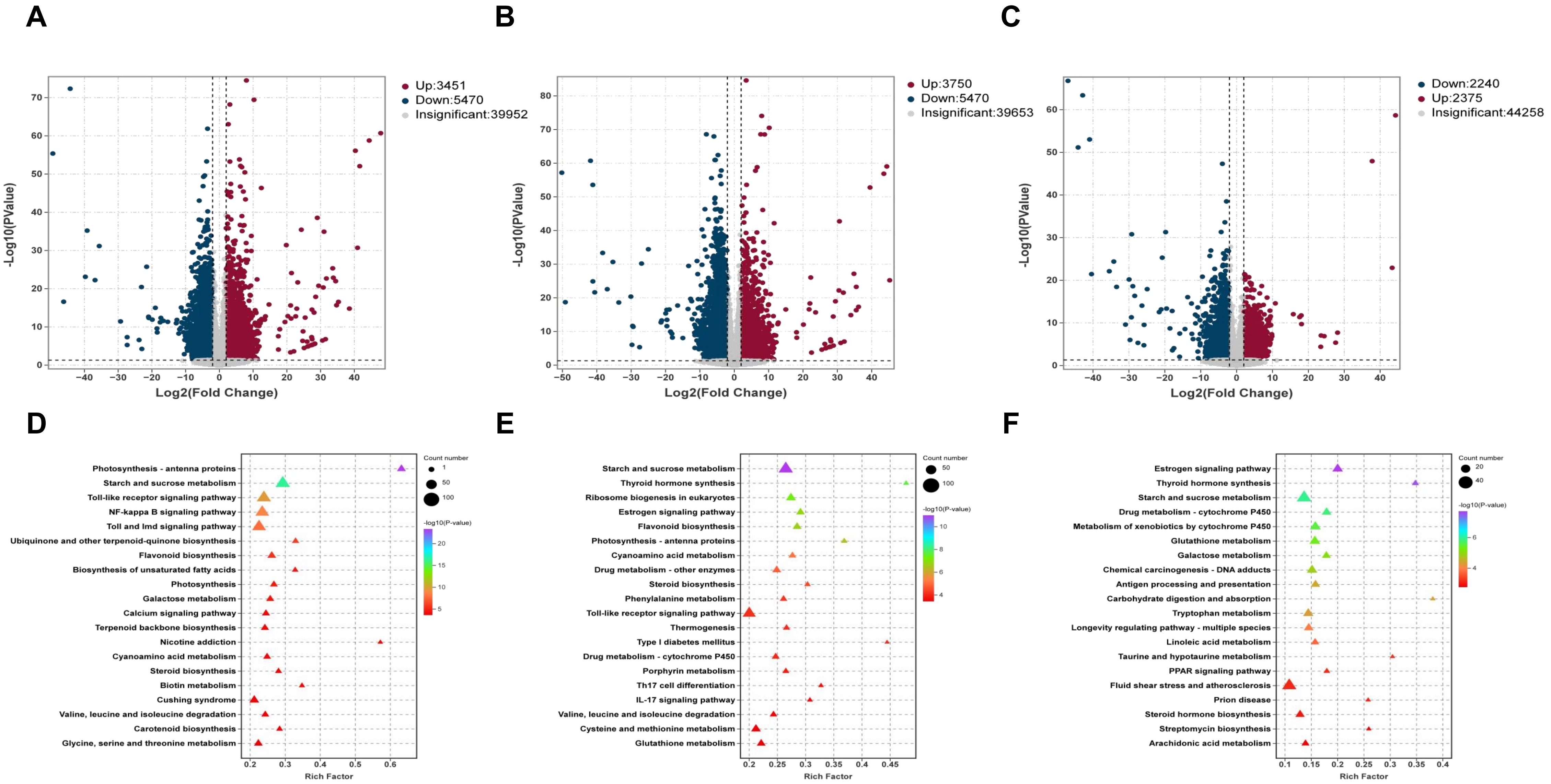

Figure S1. Differential gene expression and KEGG pathway enrichment analysis during tobacco leaf development. (A–C) Volcano plots showing differentially expressed genes (DEGs) between developmental stages (T1 vs. T2, T1 vs. T3, and T2 vs. T3). Red and blue dots represent significantly upregulated and downregulated genes, respectively (|$\log_2$FoldChange| > 1, adjusted p-value < 0.05), while gray dots indicate non-significant changes. (D–F) KEGG enrichment analysis of DEGs. Dot color represents statistical significance, dot size indicates the number of DEGs enriched in each pathway, and the rich factor on the x-axis denotes the ratio of DEGs to the total number of genes in the pathway.
